# Effects of protease-treated royal jelly on muscle strength in elderly nursing home residents: A randomized, double-blind, placebo-controlled, dose-response study

**DOI:** 10.1038/s41598-017-11415-6

**Published:** 2017-09-12

**Authors:** Ge Meng, Honglei Wang, Yinghua Pei, Yanmei Li, Hongmei Wu, Yanqi Song, Qi Guo, Hui Guo, Shinobu Fukushima, Tomoki Tatefuji, Jiazhong Wang, Huanmin Du, Qian Su, Wen Zhang, Suxing Shen, Xiuyang Wang, Renwei Dong, Peipei Han, Tatsuma Okazaki, Ryoichi Nagatomi, Jianhua Wang, Guowei Huang, Zhong Sun, Kun Song, Kaijun Niu

**Affiliations:** 1 Nutritional Epidemiology Institute and School of Public Health, Tianjin Medical University, Tianjin, China; 2 Tianjin University of Traditional Chinese Medicine, Tianjin, China; 3 Department of Rehabilitation and Sports Medicine, Tianjin Medical University, Tianjin, China; 4 Department of Rehabilitation Medicine, Cardiovascular Clinical College of Tianjin Medical University, TEDA International Cardiovascular Hospital, Tianjin, China; 5 Institute for Bee Products & Health Science, Okayama, Japan; 6 Department of Respiratory Medicine, Tohoku University Hospital, Sendai, Japan; 7 Division of Biomedical Engineering for Health & Welfare, Tohoku University Graduate School of Biomedical Engineering, Sendai, Japan

## Abstract

Although we have found that protease-treated royal jelly (pRJ) benefit for the skeletal muscle mass and strength in the aged animals, the potential beneficial effects have not been evaluated in humans. The aim of this study was to determine whether pRJ intake had beneficial effects on muscle strength in elderly nursing home residents. One hundred and ninety-four subjects enrolled into this multicenter, randomized, double-blind, placebo-controlled study. Subjects received either placebo(Group 1), pRJ 1.2 g/d(Group 2), or 4.8 g/d(Group 3). Data through 1 year are reported for 163 subjects. The primary outcome measure is handgrip strength. Secondary outcomes include several physical performance tests (six-minute walk test, timed up and go test, and standing on one leg with eyes closed). The dropout rate was 16.0%. The means (95% confidence interval) of change in handgrip strength for placebo, low-dose, and high-dose groups are −0.98(−2.04,0.08), 0.50(−0.65,1.65) and 1.03(−0.37,2.44) kg (*P* = 0.06, *P* for trend = 0.02), respectively. No significant effects of the interventions were observed for physical performances. These findings suggest that pRJ treatment might not improve, but rather attenuate the progression of decrease in muscle strength in elderly people. In addition, we have not found that pRJ intervention can achieve improvement or attenuating the decrease in physical performance.

## Introduction

The number of people aged 65 or older is projected to grow from an estimated 524 million (8 percent of the world’s population) in 2010 to nearly 1.5 billion (16 percent of the world’s population) in 2050^1^. Aging is associated with degenerative loss of skeletal muscle mass, quality and function^2, 3^. An accelerated decline in muscle mass and strength with aging (a condition known as age-related sarcopenia) is associated with frailty, functional limitations in daily living, disabilities, and, eventually, a higher mortality rate in late life^4–6^. As the aging of the population accelerates, methods prevent the loss of muscle mass and function become an important public health concern.

Satellite cells are resident myogenic progenitors in skeletal muscles. They play a significant role in skeletal muscle growth and regeneration^7^. Age-related functional decline and decreases in the number of satellite cells contribute to loss of skeletal muscle mass and function in elderly population^8^. Royal jelly (RJ), a nutrient-rich natural health food secreted by young worker bees^9^, has numerous potential pharmacological capacities, such as prolonging the life-span^10, 11^, antioxidant, and anti-inflammatory effects^12, 13^, implying that RJ might have a beneficial effect on the prevention of aged-related muscle loss and function decline through improving the functions of satellite cells^14, 15^.

In a previous study, we found that *in vitro*, RJ and protease-treated RJ (pRJ, a developed food, which has better digestion and absorption than RJ) increased the cell proliferation rate, promoted the cell differentiation, and activated Akt signaling pathway compared to controls in isolated satellite cells from aged mice; RJ and pRJ treatment increased the muscle weight, handgrip strength (Wire Hang Test), regenerating capacity of injured muscles, and serum insulin-like growth factor-1 (IGF-1) levels compared to controls in aged mice^16^. Although these findings suggested that RJ or pRJ treatment may reduce declines in muscle mass and function in aged animals, the benefits to humans have not been evaluated. Therefore, we performed this study to explore the effect of pRJ treatment on muscle strength in elderly nursing home residents.

## Results

pRJ is a developed product which uses proteolytic enzymes to break down RJ proteins into smaller peptide molecules or amino acids. The vitamin, mineral, and 10-Hydroxydecylenic acid components of pRJ are shown in Table 1. Table 2 shows the components included in the placebo and pRJ tablets (400 mg pRJ per tablet).

**Table 1:** The vitamin and mineral composition of royal jelly products (mg per 100 g).

Components	Protease-treated royal jelly
Minerals	
Sodium	2050
Phosphorus	580
Iron	2.80
Calcium	44.8
Potassium	766
Magnesium	74.3
Copper	0.91
Zinc	5.62
Manganese	0.17
Selenium	0.006
Vitamins	
Thiamine	0.84
Riboflavin	1.92
Vitamin B6	0.63
α-tocopherol	>0.1
Folic acid	0.06
Pantothenic acid	14.5
Biotin	0.07
Inositol	41
Niacin	15.4
Choline	480
10-Hydroxy-2-decenoic acid	3.64 g
10-Hydroxydecanoic acid	0.85 g

**Table 2:** Components of placebo and royal jelly tablets (12 tablets).

Components	Placebo	Low-dose pRJ	High-dose pRJ
**(Uncoated tablets, mg)**			
pRJ lyophilized powder	0	1200	4800
Dextrin	3528	2646	0
Cellulose	2234	1866	761
Sucrose fatty acid esters	118	119	124
**(Coating agent, mg)**			
Shellac	164	154	123
Zein	64	61	54
Caramel	42	39	30
L-arginine	26	25	20
Glycerin	1	1	1

pRJ, protease-treated royal jelly.

**Figure 1:**
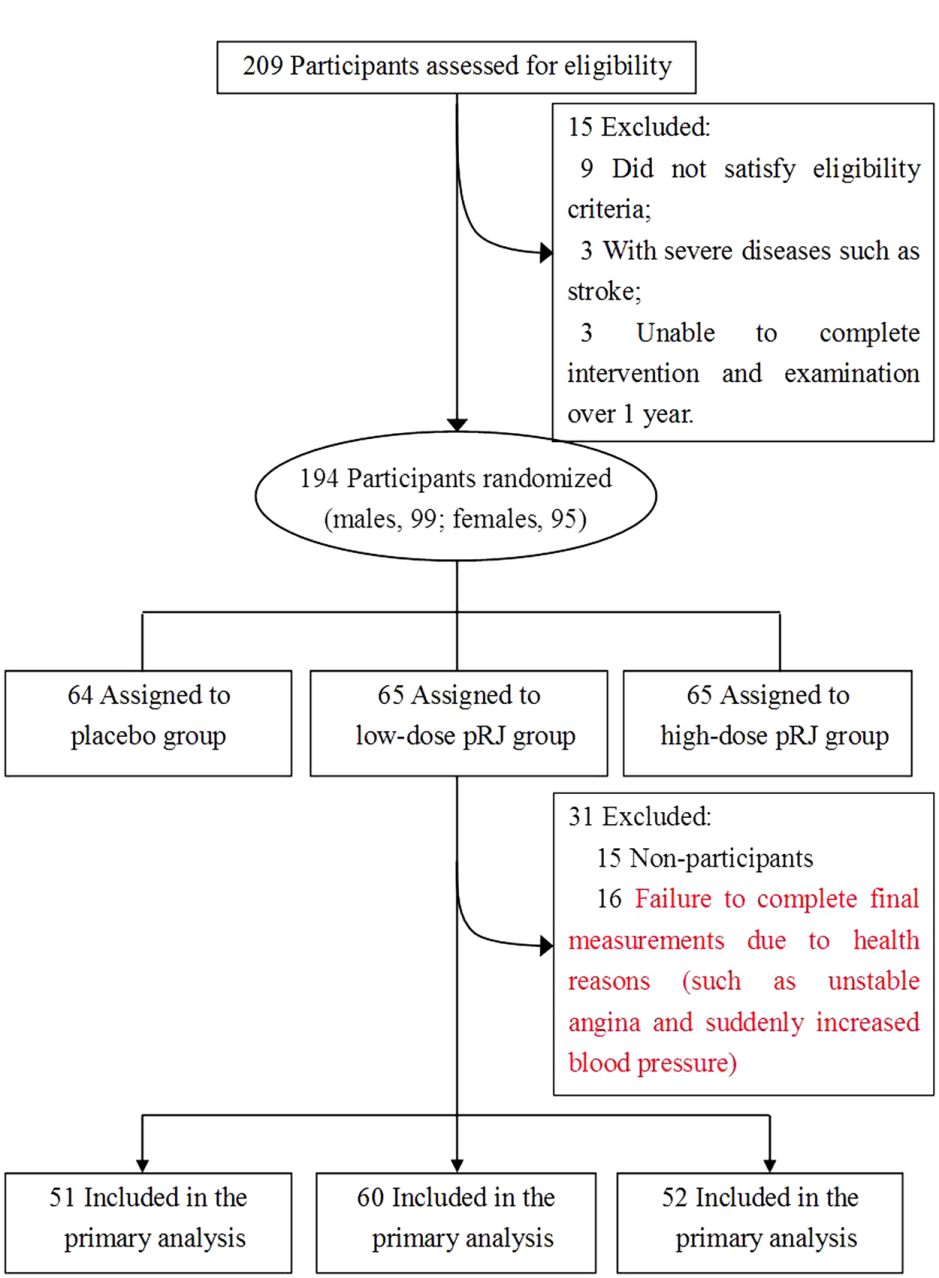
Screening, randomization, and analysis of the participants. pRJ indicates protease-treated royal jelly.

Figure 1 shows the flow of participants from the time they were screened to the end of the study, i.e., after 12 months. Ninety-nine males and ninety-five females met the inclusion criteria and consented to participate in the trial. Both the intervention and placebo groups were tolerated well by the study participants, and no treatment related adverse events occurred. Thirty-one subjects were excluded from the primary analysis (16 were not assessed during the final measurement because of healthy problems, such as heart attack and suddenly increased blood pressure; 15 dropped out of the study). The relative proportions of drop-out versus failure to complete final measurements due to health reasons for the placebo, low-dose pRJ and high-dose pRJ groups were 53.8% (7/13), 60.0% (3/5) and 46.2% (6/13), respectively. The χ^2^ test showed no observed significant differences among groups (*P* = 0.85). The total dropout rate was 16.0%. There were no significant differences in any of the baseline variables of the dropout subjects and the subjects who completed the study. For the subjects who completed the study, compliance, defined as the number of oral tablets taken, had a median (interquartile range) value of 5.5 (4.5–6.2) days (12 tablets per day) per week. Moreover, all participants completed the first three months of the study and exhibited a median value of 6.0 (interquartile range 5.0–7.0) days per week. There were no significant differences in compliance between the intervention and placebo groups.

### Characteristics of the participants

Analyses included 194 participants (99 males and 95 females; mean ± SD age: 78.5 ± 7.5 years). At baseline, no significant differences among the low-dose pRJ, high-dose pRJ and placebo groups were observed (Table 3).

**Table 3:** Baseline characteristics of trial participants^1^.

	Placebo	Low-dose pRJ	High-dose pRJ	*P* values^2^
No. of subjects	(n = 64)	(n = 65)	(n = 65)	
Age (year)	78.3 (76.8, 79.8)^3^	79.5 (78.0, 81.0)	78.0 (76.5, 79.6)	0.37
BMI (kg/m^2^)	28.2 (24.9, 31.5)	26.1 (22.8, 29.4)	26.3 (23.1, 29.6)	0.63
SBP (mmHg)	147.6 (141.9, 153.3)	151.2 (145.5, 156.9)	147.9 (142.2, 153.6)	0.62
DBP (mmHg)	73.6 (70.9, 76.3)	73.5 (70.8, 76.2)	76.2 (73.5, 78.9)	0.29
TC (mmol/L)	5.67 (5.43, 5.91)	5.59 (5.35, 5.83)	5.46 (5.23, 5.70)	0.47
TG (mmol/L)^4^	1.39 (1.22, 1.57)	1.28 (1.13, 1.45)	1.46 (1.30, 1.67)	0.31
HDL (mmol/L)	1.27 (1.19, 1.34)	1.35 (1.27, 1.43)	1.27 (1.20, 1.35)	0.23
LDL (mmol/L)	3.55 (3.33, 3.77)	3.47 (3.26, 3.68)	3.28 (3.07, 3.49)	0.21
FBS (mmol/L)	6.07 (5.73, 6.41)	5.77 (5.45, 6.10)	5.98 (5.65, 6.31)	0.44
HbA1c (%)	5.72 (5.53, 5.91)	5.74 (5.56, 5.92)	5.66 (5.48, 5.84)	0.81
History of CVD (%)	50.0	46.2	46.2	0.97
Self-rating depression Scale	35.5 (33.4, 37.5)	37.1 (35.1, 39.1)	35.6 (33.6, 37.6)	0.44
Handgrip strength (kg)	22.3 (20.0, 24.5)	21.5 (19.3, 23.8)	20.6 (18.4, 22.9)	0.63
6MWT (m)	316.4 (288.5, 344.4)	319.5 (291.7, 347.2)	325.2 (297.2, 353.1)	0.91
TUGT (sec)	11.9 (10.1, 13.8)	13.1 (11.3, 15.0)	13.4 (11.5, 15.3)	0.52
Standing on one leg with eyes closed (sec)				
right leg	3.42 (2.09, 4.75)	4.52 (3.18, 5.86)	2.87 (1.55, 4.18)	0.21
left leg	3.12 (1.92, 4.30)	4.29 (3.10, 5.49)	3.53 (2.35, 4.72)	0.38
MMSE^4^	27.7 (26.6, 28.5)	26.8 (25.8, 27.7)	26.6 (25.5, 27.4)	0.26
PA (Mets × hours/week)^4^	17.1 (13.1, 22.2)	20.3 (15.6, 26.6)	18.0 (13.9, 23.3)	0.63
Smoking status (%)				
Current smoker	17.5	27.7	13.9	0.34
Former smoker	22.2	21.5	24.6	
Never smoker	60.3	50.8	61.5	
Drinking status (%)				
Current drinker	26.2	24.6	15.4	0.68
Former drinker	18.0	15.4	16.9	
Never drinker	55.7	60.0	67.7	
Fall (%)	22.2	30.8	26.1	0.56
Education level (≥senior high school, %)	50.0	55.4	41.5	0.21

^1^pRJ, protease-treated royal jelly; BMI, body mass index. SBP, systolic blood pressure; DBP, diastolic blood pressure; TC, total cholesterol; TG, triglyceride; HDL, high-density lipoprotein-cholesterol; LDL, low density lipoprotein cholesterol; FBS, fasting blood sugar; HbA1c, Hemoglobin A1c; CVD, cardiovascular diseases; 6MWT: six minute walk test; TUGT, timed up and go test; MMSE, Mini Mental State Examination; PA, physical activity; and Mets, metabolic equivalents.

^2^Analysis of variance or χ^2^ test.

^3^(95% confidence interval, all such values).

^4^Geometric mean (95% confidence interval).

### Primary endpoint: muscle strength

The group changes in muscle strength and physical performance over 12 months were shown in Tables 4 and 5. In the primary analysis (Table 4), the means (95% confidence interval (CI)) of change in muscle strength for placebo, low-dose, and high-dose groups are −0.98 (−2.04, 0.08), 0.50 (−0.65, 1.65) and 1.03 (−0.37, 2.44) kg (*P* = 0.06, *P* for trend = 0.02). Moreover, these results have remained essentially unchanged after adjustment for nursing homes. Pre protocol based (PPB) analysis was conducted in 150 compliant participants, including 75.0% of participants in the placebo group, 84.6% in the low-dose pRJ group, and 72.3% in the high-dose pRJ group (Table 5). Analysis of variance (ANOVA) showed similar results among the intervention groups and placebo group as compare to the primary analyses. There was a significant difference in the change in the muscle strength between the groups in favor of the high-dose pRJ group [−0.96 (−2.04, 0.11) kg vs. 1.21 (−0.31, 2.74) kg, *P* = 0.048].

**Table 4:** The change in muscle strength, physical performance and IGF-1 over time and the difference between the groups at baseline and after 12-month follow-up (the primary analysis)^1^.

Variables	Placebo			Low-dose pRJ			High-dose pRJ			*P* values^2^	*P* for trend^2^	*P* values^3^	*P* for trend^3^
	(n = 51)			(n = 60)			(n = 52)						
	Baseline	At 12 Months	Changes	Baseline	At 12 Months	Changes	Baseline	At 12 Months	Changes				
Handgrip strength (kg)	22.3 (20.0, 24.6)^4^	22.0 (19.2, 24.7)	−0.98 (−2.04, 0.08)	21.4 (19.3, 23.5)	22.8 (20.4, 25.1)	0.50 (−0.65, 1.65)	20.9 (18.6, 23.1)	21.9 (19.2, 24.6)	1.03 (−0.37, 2.44)	0.06	0.02	0.05	0.02
6MWT (m)	341.0 (308.4, 373.7)	335.5 (300.3, 370.7)	−5.50 (−22.7, 11.6)	339.9 (307.0, 372.9)	319.3 (289.5, 349)	−20.7 (−41.0, −0.4)	335.7 (300.7, 370.7)	323.6 (289.4, 357.8)	−12.1 (−28.8, 4.7)	0.50	0.63	0.49	0.61
TUGT (sec)	10.6 (9.10, 12.1)	12.0 (9.70, 14.4)	1.40 (0.04, 2.76)	12.2 (10.4, 13.9)	12.9 (11.0, 14.9)	0.78 (0.05, 1.52)	13.6 (10.9, 16.3)	14.1 (10.8, 17.4)	0.50 (−1.34, 2.34)	0.63	0.35	0.6	0.32
**Standing on one leg with eyes closed (sec)**													
right leg	4.08 (2.51, 5.65)	3.31 (2.48, 4.14)	−0.77 (−2.35, 0.81)	5.03 (2.86, 7.21)	4.40 (2.64, 6.15)	−0.64 (−3.09, 1.82)	3.26 (2.23, 4.30)	2.90 (2.06, 3.73)	−0.37 (−1.42, 0.69)	0.96	0.77	0.99	0.88
left leg	3.72 (2.91, 4.53)	4.35 (3.05, 5.65)	0.63 (−0.73, 1.99)	4.68 (2.70, 6.66)	4.98 (3.27, 6.69)	0.30 (−0.84, 1.43)	4.16 (2.84, 5.49)	3.43 (2.42, 4.45)	−0.73 (−2.00, 0.54)	0.29	0.14	0.24	0.11
IGF-1 (ng/ml)	97.4 (75.5, 119.4)	79.1 (71.8, 86.3)	−20.0 (−44.2, 4.20)	86.7 (77.8, 95.5)	90.8 (83.0, 98.6)	4.80 (−5.90, 15.6)	86.5 (75.2, 97.8)	85.1 (77.7, 92.6)	2.10 (−11.5, 15.8)	0.08	0.07	0.12	0.12

^1^pRJ, protease-treated royal jelly; 6MWT: six minute walk test; TUGT, timed up and go test; IGF-1, insulin-like growth factor-1.

^2^ *P* values for differences between the groups over the 12-month study period.

^3^Adjusted for nursing home.

^4^Mean (95% confidence interval).

**Table 5:** The change in muscle strength, physical performance, and IGF-1 over time and the difference between the groups at baseline and after 12-month follow-up (per protocol based analysis)^1^.

Variables	Placebo			Low-dose pRJ			High-dose pRJ			*P* values^2^	*P* for trend^2^	*P* values^3^	*P* for trend^3^
	(n = 48)			(n = 55)			(n = 47)						
	Baseline	At 12 Months	Changes	Baseline	At 12 Months	Changes	Baseline	At 12 Months	Changes				
Handgrip strength (kg)	23.5 (20.7, 26.3)^4^	22.5 (19.8, 25.3)	−0.96 (−2.04, 0.11)	22.6 (20.2, 25.0)	23.2 (20.7, 25.8)	0.61 (−0.63, 1.84)	21.4 (18.8, 24.1)	22.6 (19.7, 25.6)	1.21 (−0.31, 2.74)^5^	0.06	0.02	0.048	0.02
6MWT (m)	344.7 (312.4, 377.0)	339.6 (306.3, 372.8)	−5.10 (−22.5, 12.2)	345.5 (310.3, 380.7)	323.8 (292.2, 355.3)	−21.7 (−44.0, 0.50)	349.0 (312.6, 385.3)	335.9 (300.9, 370.8)	−13.1 (−31.2, 5.00)	0.48	0.58	0.48	0.57
TUGT (sec)	10.4 (8.90, 11.9)	11.5 (9.40, 13.6)	1.10 (−0.15, 2.34)	12.0 (10.2, 13.9)	12.9 (10.7, 15.0)	0.81 (0.04, 1.59)	13.3 (10.3, 16.2)	13.5 (9.90, 17.0)	0.19 (−1.82, 2.20)	0.64	0.35	0.63	0.34
**Standing on one leg with eyes closed (sec)**													
right leg	4.17 (2.52, 5.81)	3.32 (2.46, 4.19)	−0.84 (−2.50, 0.81)	5.23 (2.84, 7.61)	4.55 (2.62, 6.48)	−0.68 (−3.39, 2.03)	3.50 (2.36, 4.63)	3.01 (2.09, 3.93)	−0.49 (−1.67, 0.70)	0.97	0.81	0.98	0.86
left leg	3.83 (3.00, 4.67)	4.47 (3.12, 5.82)	0.63 (−0.79, 2.06)	4.80 (2.62, 6.98)	5.25 (3.38, 7.12)	0.45 (−0.79, 1.69)	4.47 (3.02, 5.92)	3.57 (2.45, 4.70)	−0.90 (−2.32, 0.53)	0.24	0.12	0.20	0.11
**IGF**-**1** (**ng/ml**)	102.9 (77.7, 128.0)	81.6 (73.0, 90.1)	−21.3 (−47.7, 5.14)	88.0 (79.1, 96.9)	91.0 (81.5, 100.5)	3.02 (−7.79, 13.8)	87.0 (75.3, 98.8)	90.9 (81.7, 100.1)	3.90 (−10.5, 18.3)	0.08	0.06	0.12	0.09

^1^IGF-1, insulin-like growth factor-1; pRJ, protease-treated royal jelly; 6MWT: six minute walk test; TUGT, timed up and go test.

^2^ *P* values for differences between the groups over the 12-month study period.

^3^Adjusted for nursing home.

^4^Mean (95% confidence interval).

^5^ *P* < 0.05 compared with placebo group.

### Secondary endpoint: physical performances

No significant effects of the interventions in both the primary and the PPB analyses were observed for six-minute walk test (6MWT), timed up and go test (TUGT), and standing on one leg with eyes closed or adjusted for nursing homes (Tables 4 and 5).

### Effect of pRJ on serum IGF-1 levels

In the primary analysis (Table 4), the means (95% CI) of change in IGF-1 for placebo, low-dose, and high-dose groups are −20.0 (−44.2, 4.2), 4.8 (−5.9, 15.6) and 2.1 (−11.5, 15.8) (ng/ml) (*P* = 0.08, *P* for trend = 0.07). In the PPB analysis (Table 5), the means (95% CI) of change in IGF-1 for placebo, low-dose, and high-dose groups are −21.3 (−47.7, 5.14), 3.02 (−7.79, 13.8) and 3.90 (−10.5, 18.3) (ng/ml) (*P* = 0.08, *P* for trend = 0.06). Although the change was not statistically significant, serum IGF-1 levels greatly decreased within the placebo group (the mean observed change was −20.0 ng/ml, *P* = 0.10 by paired t-test; see Table 4), but not within the low-dose pRJ group (the mean observed change was 4.80 ng/ml, *P* = 0.37 by paired t-test; see Table 4) and the high-dose pRJ group (the mean observed change was 2.10 ng/ml, *P* = 0.75 by paired t-test; see Table 4).

## Discussion

We based our study on previous animal research, which showed that dietary supplementation with RJ or pRJ may reduce declines in muscle mass and function in aged mice^16^. We conducted a small-scale pilot study (8 participants per group) to examine the effect of pRJ on muscle strength and physical performance in free-living elderly people (TRIAL REGISTRATION: UMIN-CTR Identifier: UMIN000004057). Having found that the intake of pRJ (low-dose: 1.2 g/d; high-dose: 4.8 g/d) for three months modestly improved muscle strength and physical performance in the elderly, this randomized, double-blinded, placebo-controlled dose-response trial was conducted. The results indicate that pRJ treatment might not improve, but rather attenuate the progression of decrease in muscle strength in elderly people. However, no significant effects of the interventions in either the primary analysis or PPB analyses were observed for physical performance.

The safety profile of pRJ was assessed in the current trial. Asthma and anaphylaxis are the most common adverse effects of pRJ intake^17^. However, we excluded participants with allergic constitution and history of asthma in the initial study design. Therefore, adverse effects such as royal jelly-induced anaphylaxis or asthmatic attack were not observed during the trial.

To the best of our knowledge, no previous studies have investigated the effects and mechanisms of RJ on muscle mass and its function. In a previous study^16^, we demonstrated that *in vitro*, pRJ increased the cell proliferation rate, promoted the cell differentiation, and activated Akt signaling pathway compared to controls in isolated satellite cells from aged mice; *in vivo*, pRJ intake increased the regenerating capacity of injured muscles and serum IGF-1 concentrations compared to controls in aged mice. Because IGF-1 has favorable effects on satellite cells and the skeletal muscles, it is considered that the increased serum IGF-1 concentrations after pRJ treatment might be one of the mechanisms of the effects of pRJ intake. The serum IGF-1 levels decline in an age-dependent manner and are a reliable index of protein-energy malnutrition in elderly^18–20^. Therefore, we considered that many nutritional components in pRJ (see the Table 1) may have partly contributed to the prevention of age-related muscle strength decline by elevating levels of IGF-1. Beyond that, the Akt signaling pathway also plays a crucial role in muscle protein synthesis and in inhibiting muscle proteolysis. The activation of Akt in myoblasts increased their cell proliferation rate and rescued them from cell death and prevented muscle atrophy including age-related muscle mass and strength decline^21, 22^. In present study, we found that the serum IGF-1 levels decline in an age-dependent manner, and although the difference was not statistically significant, the intervention groups increased more over time in IGF-1 levels than the placebo group. Based on these observations, we infer that the activation of IGF-1 and the Akt signaling pathway are the possible mechanisms to partly explain the effects of pRJ intervention on muscle strength observed in the present study. However, the Akt signaling pathway was not studied in our investigation due to the unavailability of muscle biopsies. Future studies are needed to determine whether similar mechanisms apply to humans.

Many nutritional components in pRJ – such as vitamins, minerals (Table 1), and amino acids, including leucine – might have contributed to preventing loss of muscle strength. Leucine is particularly well-established as a nutritional factor that can contribute to muscle metabolism^23^. Our previous study demonstrated that supplementation by beta-hydroxy-beta-methylbutyrate, a metabolite of leucine, contributed to preservation of muscle mass in older adults^24^. Moreover, royalactin was recently identified as a component of RJ. This substance enables a bee to develop into a queen^25^. The effect seems to be mediated by the epidermal growth factor receptor (EGFR) signaling pathway^25^. Previous studies have found that the EGFR signaling pathway plays a role in muscle growth, maintenance, and differentiation^26–28^. Therefore, our results may be partially explained by the inclusion of leucine and royalactin within pRJ. Further study is necessary to explore each component’s effect on the prevention of loss of muscle strength.

The pRJ treated groups had a higher trend of increase in muscle strength than the placebo group, but when comparing results within the same groups before and after the treatment period, muscle strength did not change in the low or high-dose pRJ treated groups, suggesting that pRJ treatment might not improve, but rather attenuate the progression of decrease in muscle strength in elderly people. On the other hand, despite the statistically significant *P* for trend, it remains unclear whether the approximately 1-kg increase in handgrip strength observed in the high-does pRJ group as compared with placebo group (see Table 4) is clinically significant. Therefore, further studies are required to evaluate whether more long-term intake of pRJ can substantially improve physical performance in elderly individuals.

In the current study, we have not found that pRJ intervention can achieve improvement or attenuating the decrease in physical performance. Nutritional supplements were widely considered as a potential intervention for decline in muscle mass and strength with aging. However, previous studies indicate that nutritional supplements have been disappointing as stand-alone treatments for sarcopenia, but that some may have benefits in combination with resistance strength training^29^. Consequently, we infer that pRJ may have a greater effect on the improvement of muscle strength and physical performance when combined with physical activity intervention. Further studies are needed to clarify whether pRJ intake combined with exercise training strategies can enhance effects on physical performance.

The major strengths of the trial are its double-blind, randomized, placebo-controlled dose-response design. To the best of our knowledge, this is the first trial primarily designed to investigate the effects of pRJ intake on age-related sarcopenia prevention in elderly nursing home residents. A limitation of the study is that muscle mass was not assessed. In addition, muscle biopsies, which can provide direct evidence in the mechanism of intervention, were not available due to difficulty obtaining the agreement of participants. Furthermore, our study was conducted in elderly nursing home residents who were active and healthy. Given this, our results may not be generalizable to general populations. Finally, since we excluded participants who did not complete the final measurements, the intention-to-treat principle cannot be adopted. This may affect the study’s internal validity.

In conclusion, the results of this 12-month trial showed that pRJ treatment might not improve, but rather attenuate the progression of decrease in muscle strength in elderly people. In addition, we have not found that pRJ intervention can achieve improvement or attenuating the decrease in physical performance. Further study will be needed to evaluate whether pRJ can enhance physical performance when combined with physical activity.

## Subjects and Methods

### Study design

We designed a randomized, double-blind, placebo-controlled, dose-response study to determine whether pRJ intake had a positive effect on muscle strength and physical performance in elderly nursing home residents. This Clinical Trial was registered with the UMIN Clinical Trials Registry on June 19, 2012. Trial ID: UMIN000008215 and can be accessed here: https://upload.umin.ac.jp/cgi-open-bin/ctr_e/ctr_view.cgi?recptno=R000009648. Ethical approval was given by the medical ethics committee of Institutional Review Board of the Tianjin Medical University with the reference number of 2010–223. All the participants gave written informed consent. The study methods and reporting were carried out in accordance with the CONSORT 2010 guidelines.

### Setting and participants

Participants were recruited for the study by the personnel department at the nursing homes between June 2012 and September 2012. The included male and female participants (aged ≥65 y) were living in three nursing homes, in Tianjin, China. A detailed personal history of physical illness and current medications was noted from “yes” or “no” responses to relevant questions, and was finally confirmed by physicians. We assessed the eligibility of 209 participants in this study. The participant selection process is described in Fig. 1. We excluded participants with a history of an allergic constitution (*n* = 2), asthma (*n* = 1), visual and hearing impairment (*n* = 3), depression (*n* = 1), dementia (*n* = 2), and severe diseases such as stroke (*n* = 3). Furthermore, participants who were unable to complete the intervention or examination were also excluded (*n* = 3). As a result of these exclusions, the final study population consisted of 194 participants.

### Randomization and intervention

A statistician was invited to generate the random allocation sequence after the baseline assessment. This statistician was not involved in the study and had no contact with the study participants. A sequence number and randomization table were randomly generated by a computer. Randomization was stratified for study center, age (65–75 y, >75 y), and sex. Participants were randomly assigned in a 1:1:1 ratio to receive daily either oral tablets containing 1.2 g/d (low-dose group, 3 pRJ and 9 placebo tablets per day), 4.8 g/d pRJ (high-dose group, 12 pRJ tablets per day) or starch placebo tablets (12 placebo tablets per day). No human studies have established the acceptable daily intake of pRJ. Therefore, we used the recommended minimum (1.2 g/d) and recommended maximum (4–5 g/d) daily intake of RJ in the Chinese market as the low-dose and high-dose, respectively. The intervention and placebo tablets were indistinguishable in taste, smell, and appearance. The trial participants, care providers, those collecting data, and those analyzing data were blinded to the intervention, and the randomization code was held securely until completion of the study. The individual participation period comprised 1year.

### Outcomes and follow-up

At baseline and the 1-year follow-up, a broad set of measurements was performed. In the current article, the primary outcome of the study is measured (handgrip strength) as well as the secondary outcomes (6MWT, TUGT, and standing on one leg with eyes closed) and adverse events. Due to reports of acute myocardial infarction and death associated with exercise testing, we obeyed advice based on exercise testing guidelines^30^ from their physicians and excluded these subjects who exhibited potential risks for acute myocardial infarction or death, such as unstable angina and elevated blood pressure while undergoing maximal muscle strength and physical performance measurements.

### Measurement of muscle strength

Handgrip strength was measured using a reliability and validity-identified electronic dynamometer (EH101; CAMRY, Guangdong, China). Dynamometer width was adjusted for optimal fit for each participant. Participants were instructed to stand upright and with the dynamometer beside but not against their body. Participants were asked to perform maximum force trial. The measurements were repeated 2 or 3 times for each hand, and the highest score was recorded in kilograms. The highest reading from either hand was used as the final score in this study.

### Physical performance tests

Physical performance was measured with three tests: 6MWT, TUGT, and standing on one leg with eyes closed. The physical performance tests were measured by a well-trained physiotherapist as follows:

6MWT^31^: Subjects underwent a practice walk at baseline to eliminate any learning effects associated with the test. Subjects were instructed to walk back and forth along a 12 m corridor for 6 min. They were allowed to stop if limited by symptoms, but were encouraged to resume the test when possible. Two standard phrases were used to encourage subjects, “You are doing well” and “Keep up the good work”. The distance walked was recorded in meters.

TUGT^32^: The participants were seated in a free-standing padded armchair (46 cm high) and asked to rise (with or without using the arm rests), walk to a mark 3 m away, turn around, and walk back to the chair and sit down. The time between rising from the seat and making contact with the back of the seat was measured in seconds. This test was repeated three times and the time of the fastest trial was recorded.

Standing on one leg with eyes closed: The standing on one leg with eyes closed measures the postural steadiness in a static position. The subjects were instructed to stand on one leg as long as possible with eyes closed, with a maximum of 60 seconds for each trial. The procedure was explained and demonstrated, and subjects were also allowed to practice once with eyes open on each leg for 10 seconds. Three trials were performed and the time of the longest trial was recorded.

### Assessment of serum IGF-1 concentration

IGF-1 was considered as a possible mechanism, but not considered as a primary outcome in the present study. The serum level of IGF-1 was measured using a chemiluminescent immunoassay kit (Snibe, Shenzhen, China) according to the manufacturer’s instructions. The detection limit of this assay is 5.0 ng/ml, intra- and inter-assay coefficient of variation were 6.4% and 12.1%, respectively.

### Compliance

Every 3 months, new tablets were sent to the participants, and they were requested to return any remaining tablets. Participants were defined as compliant when at least 80% of the tablets had been taken during the intervention period, as indicated by the returned tablets.

### Adverse events

Adverse events (i.e., ill health–related conditions) were recorded by participants on the study calendars. In addition, all events reported to the study team by phone or otherwise were recorded. If a participant died during the study period, this was reported by relatives.

### Baseline characteristics

Blood pressure (BP) was measured twice from the upper left arm using an automatic device (Andon, Tianjin, China) after 5 minutes of rest in a seated position. The mean of these 2 measurements was taken as the BP value. Fasting blood sugar (FBS) was measured by the glucose oxidase method, triglycerides (TG) were measured by enzymatic methods, low-density lipoprotein cholesterol (LDL) was measured by the polyvinyl sulfuric acid precipitation method, and high-density lipoprotein cholesterol (HDL) was measured by the chemical precipitation method using reagents from Roche Diagnostics on an automatic biochemistry analyzer (Roche Cobas 8000 modular analyzer, Mannheim, Germany).

The anthropometric variables (height and body weight) were recorded by using a standard protocol. The sociodemographic and lifestyle variables were obtained from a questionnaire survey. Physical activity (PA) in the most recent week was assessed using the short form of the International Physical Activity Questionnaire (IPAQ)^33^. Cognitive function and depressive symptoms were tested by using the Chinese language version of the 30-point Mini-Mental State Examination (MMSE) and the Zung Self-Rating Depression Scale (SDS), respectively^34, 35^.

### Power calculation

The effect size was calculated as the difference (4.8 g/d pRJ group minus placebo group) between changes (initial minus final) in these mean values. We applied a two-tailed t-test to calculate the minimum required total sample size. The null hypothesis is that there is no difference between the high-dose pRJ group and the placebo group on average. The alternative hypothesis is that the high-does pRJ group has a different effect, on average, compared to that of the placebo group. A preliminary study indicated that the mean muscle strengths of elderly males and females were 28 and 15 kilograms, respectively (TRIAL REGISTRATION: UMIN000004057). Based on this preliminary study, we also estimated a 3% (standard deviation, SD = 1 and 2 in males and females, respectively) difference between the placebo and 4.8 g/d pRJ groups in the post-intervention muscle strength measurement. To establish statistical significance (alpha) at 5%, with a statistical power of 0.9 and assuming a dropout rate of 20%, a minimum of 30 (males) and 28 (females) participants per group was required.

### Statistical analysis

All statistical analyses were performed by using the Statistical Analysis System for Windows, version 9.1 (SAS Institute Inc., Cary, NC). For normally distributed continuous variables, the arithmetic means and SD were calculated, and for logarithmically transformed continuous variables the geometric means and SDs were computed. For a baseline comparison between the placebo and intervention groups, the χ^2^ test was used to analyze the categorical data. For comparing the continuous variables related to the basic characteristics of the three groups, ANOVA were performed. Paired t tests were performed to compare the change in the outcome variables within groups, while ANOVA was performed to compare these changes among groups. Tests for trend were conducted by using orthogonal linear contrasts. We also applied analysis of covariance (ANCOVA, Post hoc, Tukey) to evaluate the muscle strength changes for the 3 groups after adjustment for nursing homes. To assess the efficacy of the interventions, primary analyses included subjects who completed the final assessments, and PPB analyses were performed that included data only from subjects who were compliant with the study protocol. The difference in number of persons who reported at least one adverse event between treatment groups was tested by using chi square analysis. For dropouts, the time until dropout was used in these analyses. For all of the outcomes, interaction with treatments was tested for the covariates nursing homes. All *P*-values for the linear trend were calculated using the three trial group categories (1 = placebo group, 2 = low-dose pRJ group, and 3 = high-dose pRJ group). The weights for contrast were −2 (placebo group), +1 (low-dose pRJ group), and +1 (high-dose pRJ group). All the tests for statistical significances were 2 sided, and *P* < 0.05 was considered statistically significant.

### Data Availability

Data cannot be made publicly available because public availability would compromise participant privacy. For data access, researchers can contact the Nutritional Epidemiology Institute and School of Public Health, Tianjin Medical University, Tianjin, China (E-mail address: nkj0809@gmail.com or niukaijun@tmu.edu.cn).
